# Knockout of caspase-7 gene improves the expression of recombinant protein in CHO cell line through the cell cycle arrest in G2/M phase

**DOI:** 10.1186/s40659-021-00369-9

**Published:** 2022-01-11

**Authors:** Fatemeh Safari, Bahman Akbari

**Affiliations:** 1 Diagnostic Laboratory Sciences and Technology Research Center, School of Paramedical Sciences, Shiraz University of Medical Sciences, Meshkinfam Ave, Shiraz, Iran; 2 Clinical Neurology Research Center, Shiraz University of Medical Sciences, Shiraz, Iran; 3 Department of Medical Biotechnology, School of Medical Sciences, Kermanshah University of Medical Sciences, Kermanshah, Iran

**Keywords:** Caspase-7, Caspase-3, CRISPR/Cas9, Recombinant protein, Cell cycle, Chinese hamster ovary cell line

## Abstract

**Background:**

Chinese hamster ovary cell line has been used routinely as a bioproduction factory of numerous biopharmaceuticals. So far, various engineering strategies have been recruited to improve the production efficiency of this cell line such as apoptosis engineering. Previously, it is reported that the caspase-7 deficiency in CHO cells reduces the cell proliferation rate. But the effect of this reduction on the CHO cell productivity remained unclear. Hence, in the study at hand the effect of caspase-7 deficiency was assessed on the cell growth, viability and protein expression. In addition, the enzymatic activity of caspase-3 was investigated in the absence of caspase-7.

**Results:**

Findings showed that in the absence of caspase-7, both cell growth and cell viability were decreased. Cell cycle analysis illustrated that the CHO knockout (CHO-KO) cells experienced a cell cycle arrest in G2/M phase. This cell cycle arrest resulted in a 1.7-fold increase in the expression of luciferase in CHO-KO cells compared to parenteral cells. Furthermore, in the apoptotic situation the enzymatic activity of caspase-3 in CHO-KO cells was approximately 3 times more than CHO-K1 cells.

**Conclusions:**

These findings represented that; however, caspase-7 deficiency reduces the cell proliferation rate but the resulted cell cycle arrest leads to the enhancement of recombinant protein expression. Moreover, increasing in the caspase-3 enzymatic activity compensates the absence of caspase-7 in the caspase cascade of apoptosis.

## Background

CHO cell line is the most common mammalian expression system vastly used for the production of therapeutics [1, 2]. This host cell is the cellular factory of about one third of all biopharmaceutical products approved by FDA since 1982 [3]. Therefore, enhancing the yield of this expression system is greatly in consideration. Responding market demands for biologicals, CHO cells must be cultured in huge densities in large bioreactors. Culture in huge density leads to environmental perturbations and cell stress due to the limitation of oxygen and nutrients as well as the accumulation of toxic metabolites. Intensive and continuous stress induces cell death employing one of these two pathways including apoptosis (programmed cell death) and passive cell death called necrosis [4]. Almost all apoptosis mediating death pathways lead to the activation of specific downstream caspase proteins. Caspases are classified into two classes composed of executor caspases (3, 6 and 7) and initiator caspases (8, 9, 10 and 12). The activation of caspase-3 and 7 by initiator caspases allows the cleavage of various substrates and triggers morphological and biochemical characteristics of apoptosis such as the release of phosphatidylserine, condensation of nuclei and fragmentation of genomic DNA [5]. Recent reports showed that caspase-7 plays a role in ROS production and cell detachment [6]. In addition to apoptosis, caspase-7 has also non-apoptotic roles, for instance in cell cycle progression. To this end, it has been reported that the proteolytic activity of caspases affects the cell cycle proteins as their substrates [7]. Caspase-mediated cleavage of cell cycle proteins causes the activation and/or translocation of these proteins such as retinoblastoma (Rb) which is cleaved by caspase-3 and -7 [8]. Following this cleavage, truncated Rb joins cyclin D3 and reduces E2F1 transcriptional activity [9]. In our recent research, caspase-7 deficient CHO cells were generated by using CRISPR/Cas9 system [10, 11]. HITI technology was employed for silencing caspase-7 by simultaneous knockout/knock-in which facilitated the selection of knocked out clones [12]. Further, results of this study demonstrated that caspase-7 deficiency had an adverse effect on cell cycle progression resulting in the reduction of cell proliferation [13]. However, the mechanism of this event still remains unclear. Accordingly, in the study at hand, we aimed to identify the mechanisms by which caspase-7 affects cell growth, cell viability and cell cycle progression and investigate the effect of caspase-7 silencing on the expression yield of recombinant protein.

## Results

### Caspase-7 deficiency reduces cell growth and cell viability

**Fig. 1:**
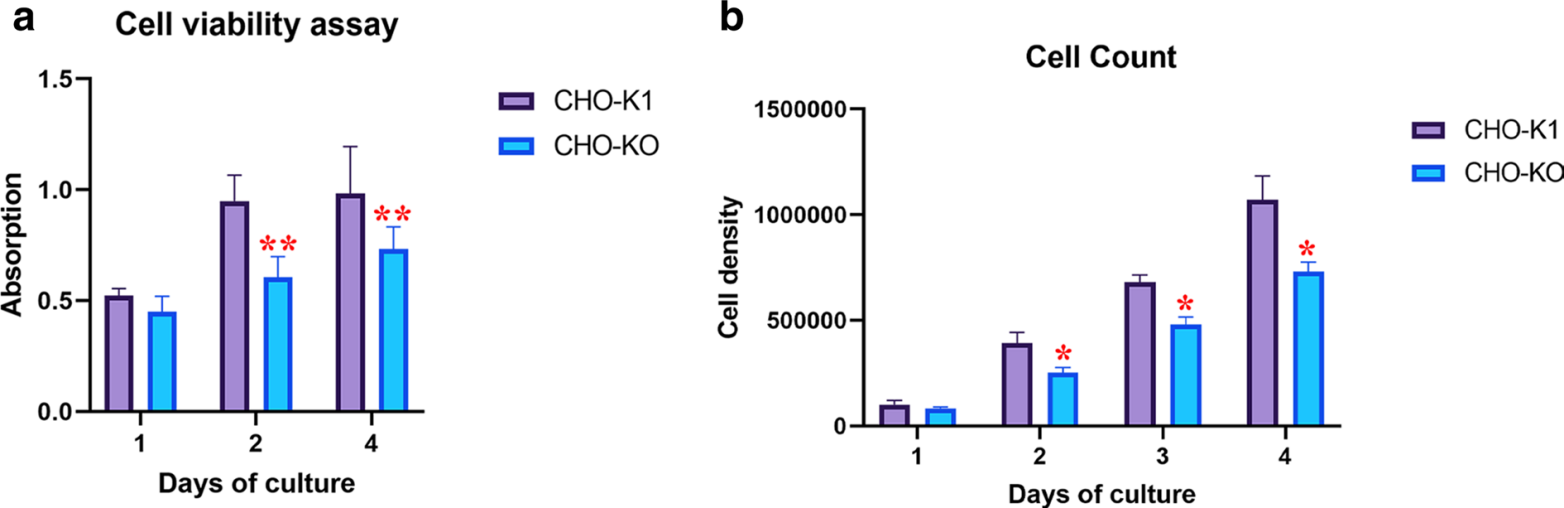
Growth dynamics and cell viability: **a** Comparison of cell viability between CHO-K1 and CHO-KO cells during 24, 48 and 96 h. **b** Comparison of cell counting between CHO-K1and CHO-KO cells during 24, 48, 72 and 96 h. Results represent the average of three analyses (n = 5), and error bars represent the standard deviation (p < 0.05). (*:> 0.05, **:> 0.01, ***:> 0.001, ****:> 0.0001)

Cell doubling time (T2) is an important parameter used for illustrating the dynamics of cell clone development which shows the average time between cell divisions. T2 is measured typically by the observation of changes in the cell density during the time [14]. Growth characteristics of native CHO-K1 and CHO-KO cells are shown in Fig. 1a, b. The growth rate of these two cell lines was comparable for 4 days, with average doubling times of 20 h for the native CHO-K1 cell line and 26 h for the CHO-KO cells.

### Caspase-7 silencing leads to cell cycle arrest in G2/M phase

**Fig. 2:**
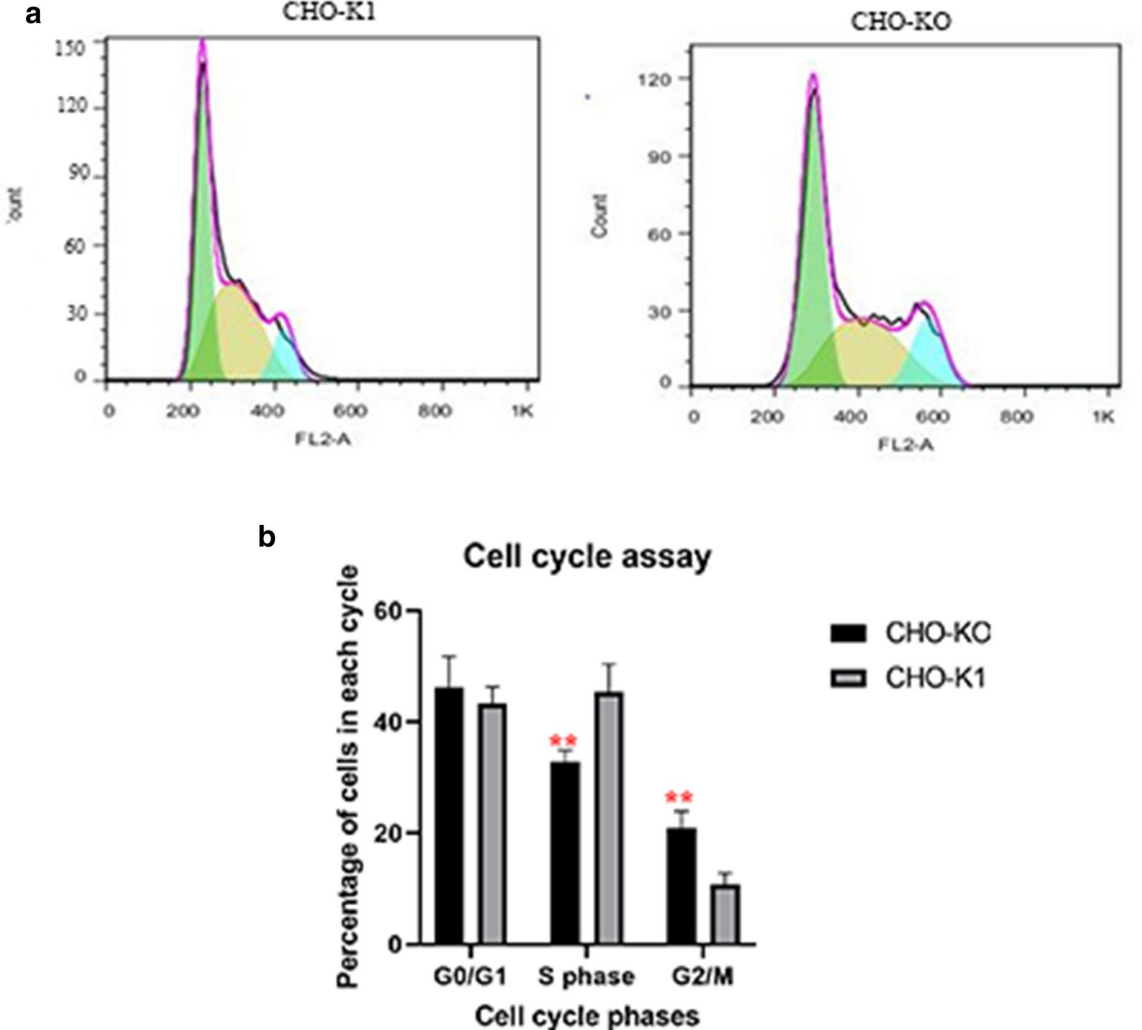
The effect of CASP7 knockout on cell cycle progression by FACS‐based, propidium iodide cell cycle analysis. **a** CHO-K1 cells, **b** CHO-KO cells. **c** Percentages of cells in the G1, S, or G2/M in asynchronous cultures calculated using FlowJo software. Results represent the average of three analyses (n = 3), and error bars represent the standard deviation (p < 0.05). (*:> 0.05, **:> 0.01, ***:> 0.001, ****:> 0.0001)

Proliferation in mammalian cells is regulated by a group of checkpoints which together form the cell cycle. These checkpoints control the events needed for accurate cell division and prevent the occurrence of each step prior to the completion of preceding steps [15]. Flow cytometric cell cycle analysis on asynchronous cells showed that compared with the native CHO-K1 cell line, the CHO-KO cell line had a 10% higher proportion of cells in the G2/M phase and a 3% higher proportion of cells in the G0/G1 phase (Fig. 2).

### Caspase-7 silencing increases the expression of luciferase and JRed in the CHO-KO cell line

**Fig. 3:**
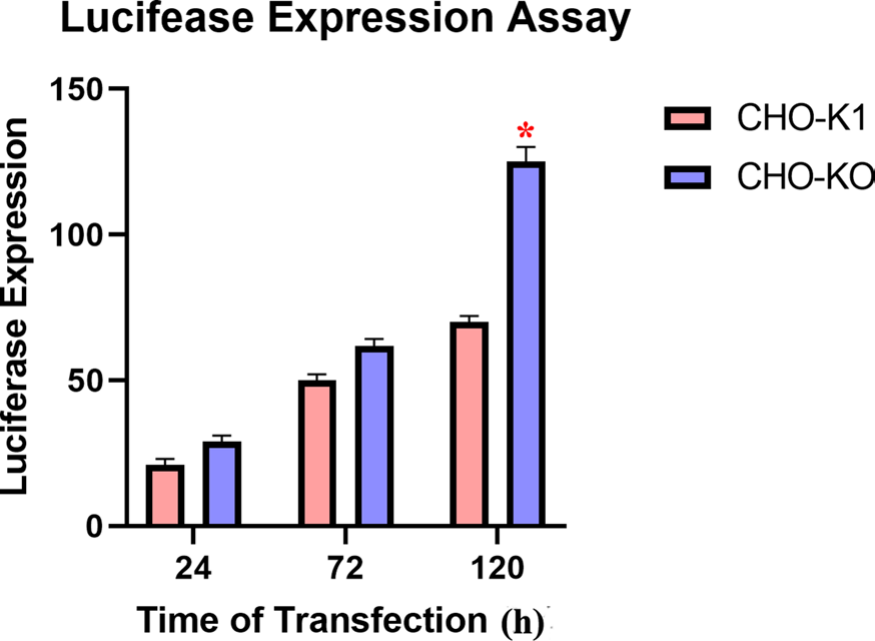
luciferase expression assay; Graph is representative of three separate production experiments during 24, 72 and 120 h after transfection, and error bars represent the standard deviation (p < 0.05). In this assay each experiment was repeated 3 times and n = 3, (*: > 0.05, **: > 0.01, ***: > 0.001, ****: > 0.0001)

**Fig. 4:**
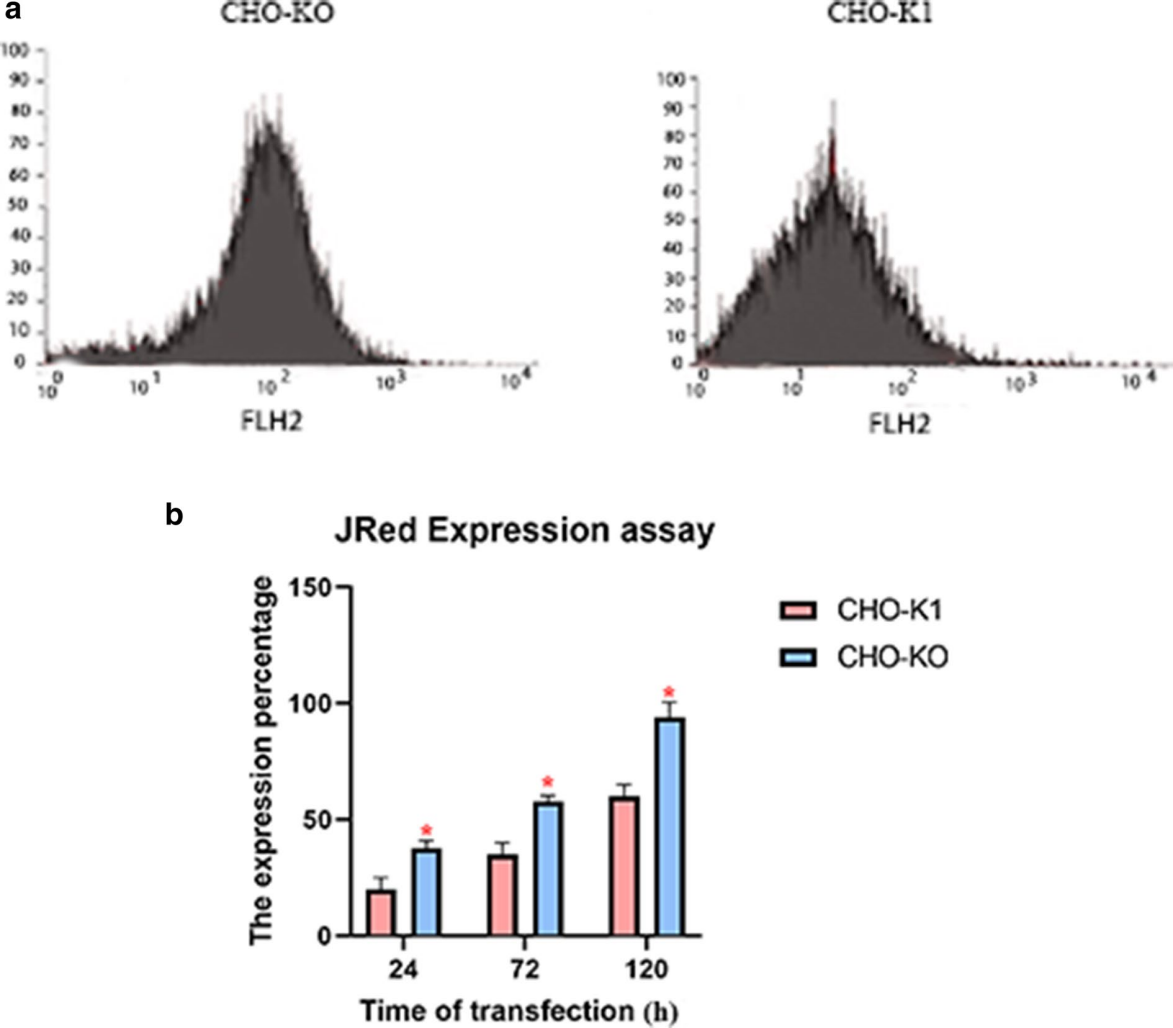
JRed expression assay; **a** The histogram of JRed expression in CHO-KO and CHO-K1 cells after 120 h of transfection, **b** the chart containing the percentage of JRed expression in CHO-KO and CHO-K1 cells after 24, 72 and 120 h after transfection. Findings represented the analysis of 3 experiments and n was 3. Error bars represent the standard deviation (p < 0.05). (*:> 0.05, **:> 0.01, ***:> 0.001, ****:> 0.0001)

The effect of caspase-7 silencing on the constitutive expression of recombinant luciferase is shown in Fig. 3. Compared with the native CHO-K1 cell line, the caspase-7 deficient clone represented an average of 1.7-fold increase in specific luciferase expression. In addition, the expression of JRed was increased by up to 1.5-fold in CHO-KO cells compared to CHO-K1 cells (Fig. 4).

### Caspase-3 compensates the absence of caspase -7

**Fig. 5:**
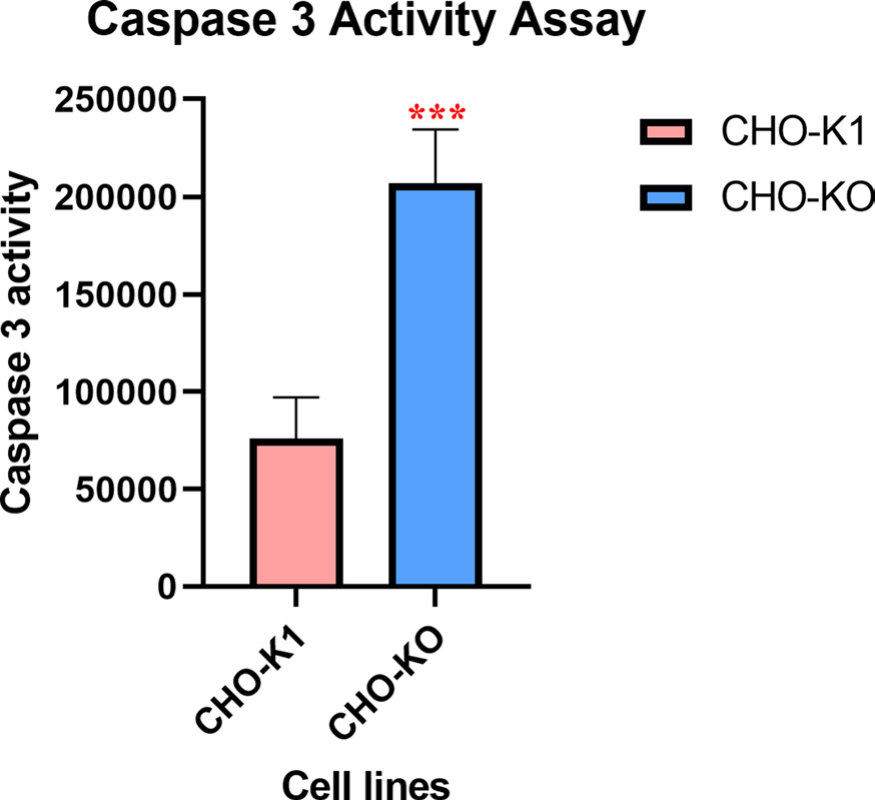
Caspase 3 activity assay; this chart represents the caspase 3 activity in lysate of CHO-KO and CHO-K1 cells undergoing the apoptosis mediated by NaBu (11 mM). Findings display the average of three analyses (n = 3), and error bars represent the standard deviation (p < 0.05). (*:> 0.05, **:> 0.01, ***:> 0.001, ****:> 0.0001)

Caspase-3 is a critical player of typical morphological and biochemical changes of cells undergoing apoptosis such as nuclear fragmentation. Findings of this assay showed that caspase-3 activity had increased by up to threefold in CHO-KO cells undergoing apoptosis mediated by NaBu in comparison with CHO-K1 cells (Fig. 5). This result represented that caspase-3 had compensated for the absence of caspase-7 in the progression of apoptosis.

## Discussion

Numerous approaches and strategies have been used for large scale and cost-effective production of biopharmaceuticals in CHO cell line to increase the production of high-quality therapeutics. These techniques include the improvement of culture media and additive supplements [16], selection of high-producing clones [17], optimization of transcriptional activity through vector engineering [18], and gene overexpression or silencing [19] using cell line engineering [1, 20]. Apoptosis engineering by overcoming the cell death and enhancing the time integral of the viable cell concentration has been one of the important strategies which increases the product yields [4].

Apoptosis has been extensively investigated in CHO cells using strategies such as slowing down or inhibiting apoptosis for prolonged culture longevity. Along with caspases as mediators of apoptosis, the Bcl-2 family including pro- and anti-apoptotic members plays a critical role in the fate of apoptosis by adjusting the release of cytochrome *c* in the mitochondrial membrane [21]. Hence, numerous studies have primarily been investigating the overexpression of anti-apoptotic members of the Bcl-2 family and the down-regulation of caspases to impede or delay apoptosis [22].

Caspases-3 and 7 have been identified as major mediators of apoptosis progression [2]. As well as caspase-3, caspase-7 is an executioner caspase involved in cleaving downstream substrates such as PARP [23]. During apoptosis, caspase-7 is activated by initiator caspases via proteolytic activity at Asp23, Asp198, and Asp206, which leads to the production of the mature caspase-7 subunits [5]. Like caspases-2 and -3, caspase-7 also cleaves substrates using DEVD sequences as recognition sites [24].

It is reported that Co-down-regulation of both caspase-3 and caspase-7 by using siRNA in CHO cells enhanced the cell viability and increased culture longevity [25]. But we reported that CRISPR system mediated caspase-7 silencing in CHO cell leading to a reduction in cell proliferation. It seems that cell cycle arrest is the cause of decreasing cell proliferation which is due to the non-apoptotic roles of caspase-7. Findings of this research represented that the doubling time of CHO-KO had increased 6 h which was related to cell cycle arrest in G2/M phase. It is worth mentioning that controlling cell proliferation by manipulating cell cycle in recombinant cell lines is in the context of biotechnology and leads to an increase in the expression of heterologous proteins. Growth arrest can be induced in both G1/G0 and G2/M checkpoints but arrest induction is more common in G1/S phase [26].

In line with our findings, a series of Hashimoto and his colleagues’ data displayed that caspase-7 involved in the cell cycle regulation at mitotic phase. They reported that caspase-3, caspase-7, caspase-8 and caspase-9 were activated in the cells in an apoptosis-independent manner. In addition, it was identified that target proteins of caspases during apoptosis have function in cell cycle development. For instance, p21 and p27, cyclin E and Rb (Cdk inhibitors) regulate the progression of cell cycle from G1 to S phases. Moreover, it seems that Bub1, Scc1/Rad 21, BubR1, INCENP and CENP-C are contributed to M phase progression [27–30]. These proteins are necessary for cell cycle checkpoints; hence the dysfunction of these proteins may lead to tumorigenesis [31–33]. Activation of caspases in cancer cells may cause the loss of cell cycle checkpoints and increase the proliferation of these tumor cells. As cell cycle assay using PI staining does not differentiate in G2 and M cell cycle phases and also the difference between the essence of cancer and normal cells, findings of our study elucidate the role of caspase 7 in targeting proteins required for G2/M progression.

Results of this study showed that the expression of luciferase in CHO-KO cells was 1.7 times more than parenteral CHO-K1 cells. This event may be due to the arrest that occurred in G2/M phase of cell cycle. The higher JRed expression (1.5-fold) in CHO-KO cells compared to CHO-K1 cells also confirmed the primary findings. In line with this evidence, Abaandou et al. reported that the caspase 8‐associated protein 2 (CASP8AP2) gene silencing improved recombinant protein expression in HEK293 cells by inducing cell cycle arrest in G0/G1 checkpoint [34]. They demonstrated that silencing CASP8AP2 lead to the enhancement of luciferase and SEAP production by up to 7- and 2.5-fold respectively. This event may be due to the cell cycle arrest in G0/G1 checkpoint. As in G0/G1 phase cells represent higher metabolic activity, inducing growth arrest at this phase is a known strategy for enhancing productivity of mammalian cell lines [35–37]. In this phase, the expression of genes involved in ribosome biosynthesis and protein translation increases [38–40]. Therefore, the difference between the protein expression in CASP8AP2 knockout HEK cells and CASP7 deficient CHO cells may associate with the difference in the phases of cell cycle arrest.

Caspase-3 and caspase-7 are responsible for the majority of cleavages that occur during apoptosis [41]. It was believed that in apoptosis the cleavage-specificity profiles of caspase-3 and caspase-7 were redundant [42, 43]. However, recent studies have reported that caspase-3 and caspase-7 must have definite functions. This data relies on the findings that showed mice deficient in these caspases represented different phenotypes [23, 44]. It has been suggested that caspase-3 is the major mediator of apoptosis, while caspase-7 plays other roles [23]. The result of this study revealed the dominancy of caspase-3 in apoptosis, because in the absence of caspase-7 the enzymatic activity of caspase-3 increased which led to apoptosis progression and cell death. This finding is confirmed by other studies reporting that caspase 3 and caspase 7 are similar structurally and caspase-3 might compensate for the lack of caspase-7 [45, 46].

## Conclusions

Taken together, results of this study suggests that the caspase-7 deficiency may lead to the increase in recombinant protein expression. Although this enhancement is not significant due to the reduction of cell proliferation, it may be helpful in small scale protein production. As fundamental research, finding of this study clears the dominant role of caspase-3 in progression of apoptosis in the lack of caspase-7 in CHO cells; which approved the previous data in this case.

## Methods

### Cells and culture conditions

CHO-K1 and CHO-KO cells were maintained in RPMI medium supplemented with 10% fetal bovine serum as described previously [47]. Cultures were incubated at 37 °C in a 5% CO_2_ atmosphere. Pen/Strep stock (1 mg/mL) was prepared and diluted into the culture medium to a final concentration of 10 μg/mL.

### Cell doubling time and viability assay

CHO-KO and native CHO-K1 cells were plated in 96-well plates with a concentration of 5 × 10^3^ per well in quintuplicate. Cells were incubated for 24, 48, 72 and 96 h [34] and after that doubling time of cells was identified by using cell counter. For cell viability assay the medium from each well was entirely removed and replaced with 100 µL of fresh serum-free culture medium containing 5 mg/mL MTT solution. After 4 h of incubation, media was replaced by 100 µL of DMSO and mixing thoroughly by using an orbit plate shaker. Then, plates were incubated for an additional 10 min and the absorbance was read at 545 and 630 nm reference wavelengths.

### Flow cytometry analysis of the cell cycle

CHO-KO and native CHO-K1 cells were seeded in the 6-well plate by the concentration of 5 × 10^5^ cells/well. After 28 h [34], cells were fixed in 70% ethanol [48], and DNA was stained using PI in the presence of DNase-free RNase A (40 mg/mL) for 30 min at 37 °C in the dark circumstance. Cell cycle analysis was performed using flow cytometry (Beckman Coulter FC500; Beckman Coulter) according to the manufacturer’s instructions.

### Luciferase activity assay

Approximately 3 × 10^4^ CHO-KO and native CHO-K1 cells were seeded and cultivated for 24 h in each well of a 96-well plate. Then 100 ng psi-check2 vector was transfected into the CHO cells in triplicate. 24, 72 and 120 h after transfection [34], cells were lysed utilizing passive lysis buffer containing Tris/phosphate 25 mM, Triton X100 1%, Glycerol 10%, and DTT 2 mM. After that the firefly intensity was measured using GloMax Multi-detection system (Promega) in a luminometer at the excitation wave length of 520 nm.

### JRed expression assay

Approximately 3 × 10^4^ CHO-KO and native CHO-K1 cells were seeded and cultivated for 24 h in each well of a 24-well plate. Then 100 ng of pLEX-JRed vector was transfected into the CHO cells in triplicate. 24, 72 and 120 h after transfection [34], the expression of JRed was assessed using flow cytometry (Beckman Coulter FC500; Beckman Coulter).

### Caspase-3 activity assay

A caspase-3 activity fluorometric assay (*N*-acetyl-Asp-Glu-Val-Asp-7-amido-4-methylcoumarin; Sigma, A1086) was used according to the manufacturer’s protocol. CHO-KO and native CHO-K1 cells were seeded at a density of 3 × 10^4^ cells/well in 24-well plates in triplicate. After 24 h, cells were exposed to NaBu (11 mM) for 48 h [13] and then they were lysed using lysis buffer (Tris/phosphate 25 mM, Triton X100 1%, Glycerol 10%, DTT 2 mM). Samples containing equal amounts of total protein were incubated with the DEVD substrate at 37 °C for 1 h. The samples were then evaluated by measuring light absorbance at an excitation wavelength of 380 nm and emission wavelength of 440 nm in an automatic microplate reader.

### Statistical analysis

All assays were performed in triplicates and repeated. Statistical analyses of the data were done using GraphPad Prism 6.0, and the data were represented as mean ± S.E. Calculations of the level of significance (p) were done by Mann–Whitney U test, A p value less than 0.05 (p < 0.05) was considered statistically significant.

## Data Availability

The datasets used and/or analyzed during the current study are available from the corresponding author on reasonable request.

## Abbreviations

CHO: Chinese hamster ovary
CRISPR: Clustered regularly interspaced short palindromic repeats
Cas: CRISPR-associated protein
HITI: Homology independent targeted integration
EGFP: Enhanced green fluorescent protein
NaBu: Sodium butyrate
